# Developing and Maintaining Public Trust During and Post-COVID-19: Can We Apply a Model Developed for Responding to Food Scares?

**DOI:** 10.3389/fpubh.2020.00369

**Published:** 2020-07-14

**Authors:** Julie Henderson, Paul R. Ward, Emma Tonkin, Samantha B. Meyer, Heath Pillen, Dean McCullum, Barbara Toson, Trevor Webb, John Coveney, Annabelle Wilson

**Affiliations:** ^1^College of Nursing and Health Sciences, Flinders University, Adelaide, SA, Australia; ^2^College of Medicine and Public Health, Flinders University, Adelaide, SA, Australia; ^3^School of Public Health and Health Systems, University of Waterloo, Waterloo, ON, Canada

**Keywords:** trust, COVID-19, pandemic management, prevention, risk communication

## Abstract

Trust in public health officials and the information they provide is essential for the public uptake of preventative strategies to reduce the transmission of COVID-19. This paper discusses how a model for developing and maintaining trust in public health officials during food safety incidents and scandals might be applied to pandemic management. The model identifies ten strategies to be considered, including: transparency; development of protocols and procedures; credibility; proactivity; putting the public first; collaborating with stakeholders; consistency; education of stakeholders and the public; building your reputation; and keeping your promises. While pandemic management differs insofar as the responsibility lies with the public rather than identifiable regulatory bodies, and governments must weigh competing risks in creating policy, we conclude that many of the strategies identified in our trust model can be successfully applied to the maintenance of trust in public health officials prior to, during, and after pandemics.

## Introduction

The effectiveness of public health interventions is dependent on the behaviors of members of the public. Central to our argument is the premise that public trust in public health officials, their messages, and the science upon which their messaging is based, contributes to the success of public health interventions (1). In the context of COVID-19, trust in public health countermeasures is particularly important at the outset of epidemics when the public has limited knowledge about the infection and must rely on official advice, and when vaccines are not yet available (2–4). Further, the abundance of messaging and misinformation available makes it critical that credible sources, such as public health messaging, are trusted in order to counter mis- and disinformation that may be harmful (5). Indeed, the willingness to adopt preventative measures has been found to be greater when people trust government and public health officials (3). For example, trust in medical authorities has been identified as a predictor of vaccination behavior and has been shown to influence perceptions of the effectiveness of protective measures (6–8). Furthermore, trust in government has been associated with adherence to recommended protective behaviors and the intention to accept vaccination regardless of what authorities actually did to manage the risk of infection (4–6, 9). In this way, public trust is an important matter for public health efforts that seek to rapidly mobilize desirable self-protective behaviors across a population in order to reduce the spread of infectious disease and protect vulnerable populations (10–13). This recognizes that rather than being passive or neutral receivers of public health advice, the public function as active constructors of risk, and may construct risk in ways that might be perceived as irrational or ignorant by public health officials (14, 15). Public trust in government and public health authorities has an important influence over public constructions of risk and their responses to the threat of infectious disease through promoting acceptance of health information (2, 16, 17). The maintenance of the credibility of, and consequent trust in, government and public health officials as an information source is therefore, an important consideration in pandemic management.

Given the need to mount a rapid public response to counter the spread of SARS-CoV-2 and resultant coronavirus disease (COVID-19), it is not surprising that there has been a surge in calls to enhance trust in governments and health authorities (18, 19), reflecting similar calls for a greater focus on trust following the 2009 H1N1 pandemic (20–22). In reviewing such claims however, what exactly is meant by “trust” is not always clear. A conceptually useful definition of trust is “a particular level of subjective probability with which an agent [the public] assesses that another agent or group of agents will perform a particular action and in a context which affects his own action” [(23), p. 217]. Calnan and Rowe argue that the truster must have positive expectations regarding the competence of the trustee, and must regard the trustee as being concerned about, and willing to act in, the best interest of the trustee for trust to be possible (24). Critical then, in terms of fostering trust, is the need for health officials to be viewed as the experts whose intentions and actions are in the best interest of the public.

In 2016, we developed a model for maintaining and regaining trust in the food regulatory system during and after food safety incidents or scandals (25). This model was developed through a two-phase research project. The first phase involved 105 interviews with key stakeholders (food regulators, food industry representatives, and media actors) across three countries (Australia, New Zealand, and the United Kingdom). Analysis of the data resulted in the identification of ten strategies which can be used by food regulators, industry and the media to maintain and regain public trust in the food system. These strategies include: transparency; development of protocols and procedures; credibility; proactivity; put the public first; collaborate with stakeholders; consistency; education of stakeholders and the public; build your reputation; and keep your promises (25). The strategies were member-tested in phase two of the study which used an electronic survey to seek agreement with and rank the strategies identified by stakeholders (25). In a subsequent study these strategies were tested with and ranked by a representative sample of the public, with high congruence between the original model and strategies suggested and ranked by the public (26).

In the discussion that follows we define each strategy and explore its applicability to the building and maintenance of trust in public health officials during pandemics drawing upon accounts of pandemic management in the academic literature and current events. We then outline some issues to consider in applying this model to pandemic management noting the ways in which pandemic management differs from the management of food safety incidents and scandals. Our aim is to demonstrate how strategies identified in the model can be applied to trust maintenance during pandemic crises.

## Applying a Model For Developing and Maintaining Public Trust to Pandemic Management

The model identified ten strategies for maintaining and building trust in food regulation that may be adapted as a means of developing or maintaining public trust in and compliance with public health risk mitigation strategies. Transparency was the ranked as the most important strategy in maintaining trust by both key stakeholders and the public, which follows the emphasis on transparency or openness as trust-building strategies during periods of pandemic (27–29). Transparency in this context was understood as providing timely information about level of risk, communicating openly, timely and honestly with the public, substantiating claims, openness about what can be investigated and accountability when things go wrong (25). This is particularly important where difficult and disruptive actions (e.g., social distancing, closure of small businesses, postponement of non-essential medical procedures) are likely to generate controversy and strong emotional responses from the public (30), raising questions of whether risk assessment and mitigation strategies communicated by government and public health authorities should be trusted, and therefore enacted. It is important therefore, that the public receive timely and accurate information about current disease status and future disease projections as this information is essential for making sense of the level of personal risk and demonstrates the effectiveness of public health strategies (14). There is also likely to be greater compliance with precautions such as social distancing if the public understand the rationale for these strategies and have a realistic understanding of the time taken to develop other more comprehensive solutions such as vaccine development for COVID-19 (31). Maintaining transparency regarding scientific uncertainty has also been identified as an important strategy for maintaining trust within the pandemic literature (32, 33), with Holmes asserting that “scientific knowledge is always provisional and uncertain, and it will be at its most uncertain during a suspected emerging infectious disease, as new information and theories surface daily. To retain trust, spokespeople must acknowledge what they do not know” [(32), p. 356].

The development of protocols and procedures was also considered to be an important strategy for trust maintenance by both stakeholders and the public. This involves the development of crisis plans and ongoing surveillance of risk (25). Globalization and subsequent difficulties in containing infectious disease within national boundaries have contributed to ongoing preparations for an influenza pandemic by international organizations including the European Center for Disease Prevention and Control and the World Health Organization (WHO) (34, 35). The WHO adopted a governance model that involved ceding national sovereignty over public health policy to the international community and establishment of pandemic phases, although the WHO's June 2013 interim guidelines allowed for greater national flexibility in response (36). The *Australian Health Management Plan for Pandemic Influenza* is for example, based upon four phases: preparation (e.g., establishing relationships, monitoring; ensuring resources are available for rapid response); standby (e.g., communication to raise public awareness); action (e.g., health care and public health responses) and stand-down (e.g., resumption of previous activities and monitoring) (37). Legido-Quigley et al. identify eight actions taken by countries successfully managing COVID-19. These are: travel restrictions and quarantine for travelers; development of surveillance systems to test the public and trace contacts; intergovernmental co-ordination; public assistance for medical costs associated with the virus; strategies to sustain existing health services; obtaining crucial care equipment, medicines and personal protective equipment; adherence with infection control practices by health services; and management of information systems to promote information sharing (38).

Credibility as a strategy was also rated highly by stakeholders and was primarily associated with the independence of medical experts from government, what has been referred to as “epistemic authority” (39). Declining trust in public institutions has enabled the proliferation of alternative sources of information leading to potential for misinformation (5). In the United States, for example, partisan news reporting by Fox News contributed to rejection of Centers of Disease Control recommendations (40). Goldstein argues that when unpalatable public health messages need to be communicated they are better received if the message is delivered by agencies which are “independent of the organizations or individuals for whom the truths are inconvenient.” [(41), p. e13]. Following the 2009 H1N1 (swine flu) pandemic there was evidence of an observed decline in public trust in medical organizations, the government, the World Health Organization, and pharmaceutical industry in Switzerland (42). This is reflective of a broader “crisis of public trust” (20, 43) following the H1N1 pandemic where the public has become more skeptical of the real risk posed, and suspicion of hidden agendas amongst health organizations and the pharmaceutical industry (2, 17). Independence is particularly important in regions where other events (e.g., social unrest in Hong Kong) have eroded trust in government (38).

A fourth strategy is being proactive. Proactivity is associated with regular review and updating of public health advice and recommended practices as new evidence emerges during the pandemic, and prompt communication about emerging issues (25). Proactivity is related to transparency through timely information sharing, release of data set and modeling, and to the development of protocols and procedures through regular review and updating of procedures. However, maintaining proactivity in pandemic preparedness and response should be assessed against the potential risks of raising false alarms, which have been shown to contribute to a public skepticism of the real risks posed by pandemics and an erosion of trust in governments and public health (20, 44).

A fifth consideration in the model is putting the public first. Our model argues for prioritizing the public but notes that while the health and safety of the public was given high priority, agreement on the importance placed upon public values (e.g., food regulation concerns with food additives or genetically modified food) were given lower priority (25). The public's health and safety is paramount to pandemic management, but, unlike food regulation, successful management only occurs if a critical mass adopts the recommended behavioral strategies (9) such as handwashing and social distancing to reduce spreading. As such, pandemic management operates at a population level rather than at the level of individual members of public and trust that officials are acting in the interests of the public is likely to increase compliance.

Education of stakeholders and the public was also identified as important for maintaining trust in food regulation (25). Education occurs through provision of timely information in accessible formats for the public. Siegrist and Zinng identify gaps in knowledge and misconceptions about vaccination and herd immunity which are further compounded by the public needing to decide between competing information sources (45). Likewise, 77% of Republicans in recent research undertaken in the United States, believed that the media exaggerated the risks of COVID-19 (40). Trust in incorrect information or even conspiracy theories (46) may prevent adoption of preventative strategies (47, 48) and can be addressed through targeted social marketing campaigns.

It is also vital to ensure that the needs of different population groups are identified and these different groups are communicated with in ways that meet their needs. Factors (including those that are health-related) will affect how individuals and population sub-groups respond to public health communications and how willing or able they are to enact communicated risk-mitigation strategies. Identifying the needs of different population groups, and ideal ways to communicate with them in order to maximize compliance with government public health messages, is paramount and further research is needed in the context of COVID-19 (11, 30). In the context of COVID-19, this is especially relevant given that there are certain groups for example older people and those with chronic conditions, who are greater risk of experiencing complications when contracting the virus (49). Hence specific communication strategies to target these people must be developed. Hence specific communication strategies to target these people must be developed. For example, following the 2009 H1N1 pandemic in New Zealand, members of vulnerable populations expressed a need for governments and health officials to communicate specific actions that they could take to protect themselves and their families from infection, suggesting that public values of self-protection were driving behavior change amongst vulnerable groups (22). In considering the public, trust-enhancing activities must be cognizant of and respond to such public values.

Collaboration with stakeholders was identified as a further strategy to improving trust in food regulation. The primary stakeholders for food regulation are the food industry and the media which disseminates information about food safety incidents or scandals and are instrumental in building or diminishing public trust in food governance (25). The media is also an important stakeholder in pandemic management. Trust in media has also been found to be positively associated with willingness to adopt preventative measures (9). An example of the effects of such collaborative work can be found in the Chinese response to 2013 H7N9 epidemic, in which the Chinese state and media organizations worked together to provide daily updates regarding the epidemic and appropriate preventative measures (alongside the suppression of misinformation), functioning to reduce rises in social anxiety during the epidemic (50). General practitioners and other health professionals are also important stakeholders as the public often trust information received from health professionals over that received through public health campaigns (6) or other community sources (51). The government, particularly those government officials involved in disseminating public health advice, are also vital stakeholders given the way in which trust might be built or eroded depending on the conduct of these communicators (52). Governments and public health officials produce pandemic response plans which guide government action and communication to the public. Building relationships and ensuring that these groups receive timely and appropriate information may therefore, increase uptake of preventative behaviors and vaccines through ensuring consistency in messaging.

The final strategies: “build your reputation”; and “keep your promises” primarily relate to action taken between food safety incidents or scandals (25). One of the key findings from our earlier study was that trust in the food system depends on actions by food regulators between as well as during food safety incidents. The study identified communication strategies which can be used to increase awareness of, and build the reputation of regulators. These may include: fostering relationships with the media to promote rapid dissemination of information; the use of social media including twitter to promote the work of the regulators; and establishment of public committees (53). Building trust is also important before a pandemic as trust in health governance has been positively associated with uptake of behavioral recommendations (6, 9). Effective performance in management of other public health issues may increase trust in health governance prior to pandemics. Other factors important for ensuring good trust exists between the public and the government prior to pandemics are the presence of pandemic response plans and maintaining good trust during “business as usual,” or proactive communication.

## Issues to Consider in Applying the Trust Model

In the following sections, we identify issues to consider when applying the trust model to the context of COVID-19. These include ways in which pandemic management differs from food safety but also issues reacted to governance. In order to support the adaption of a model of trust-building strategies relevant to food crises to trust-building in the context of epidemics and pandemics (as crises of infectious disease), there needs to be an explicit examination of assumptions made about the concept of trust and the socio-political context of trust.

### In Who Do we Trust?

Food regulation differs from management of pandemics as food regulation is an ongoing process with an identifiable body that is responsible for food safety with established protocols for communication of information. During pandemics, responsibility for management falls to government working in consort with public health officials and the health professions. Within the pandemic-trust literature, this has contributed to a focus on trust in government (42, 50, 51), healthcare industry (42), public health organizations (6, 42, 54, 55), communicators of public health messages (22, 52, 56), and the communicated messages themselves (57). Effective pandemic management has been associated with “agreed communication strategies, [a] clear division of responsibilities” and agreed policy guidelines (35, p. 21). Difficulties can arise when/if contradictory messages are received from key players. This has been the case during the current COVID-19 pandemic in the United States. Survey research conducted in the US in February 2020 found that 69% of participants favored public health leadership (either Centers for Disease Control or National Institutes for Health) over political leadership of the pandemic response (19). The domination by the US President of daily updates and provision of information which is contrary to public health has eroded public trust in government (58). A recent poll found that both Democrats and Republicans express diminishing trust in the President with only 23% of respondents expressing high levels of trust in COVID-19 information given by the President (58). This contrasts with the Australian experience where a high proportion of Australians rate the Government's response favorably (59).

### Impact of Risk Perception

Perception of the risks posed by a pandemic has been identified as an important motivator for continued compliance with preventative measures after the initial phase of the illness (4, 60). Yet information about disease risk during pandemics is often provisional or ambiguous (13, 20, 30). Processes such as social isolation and social distancing in contrast, are associated with identifiable economic risk both for the country and for the individual. Competing risks must be balanced by government in lifting restrictions. Brown argues that this decision is often informed by political ideology rather than public health. He argues that “governments following distinctively right-leaning, economically liberal, socially conservative and individualizing policy trajectories” are more likely to adopt conservative management strategies but acknowledges that this may reflect cultural norms which promote suspicion of public intervention in the private sphere [(14), p. 3]. Regardless of underlying cause there is evidence that countries that were slow to initiate preventive measures and/or quick to remove restrictions (e.g., United Kingdom, United States, Brazil) or that introduced minimal restrictions (e.g., Sweden) have experienced higher rates of morbidity and mortality (61). Further, given that a critical mass is required for effective pandemic management, the options for individual management of risk are reduced in these settings.

### Federalism and Fragmentation

In contrast to food regulation where relationships between government are formalized, further issues may arise when co-operation across multiple levels of government, either across or within nations, occurs. Different levels of government may have different agendas with competing agendas requiring the public to choose which level of government to trust. Federalism has been associated with duplication of services, difficulties in identifying who is responsible, gaps in service delivery and shifting of responsibility across levels of government (62). In Australia for example, the provision of health care services lies with state governments, while the Commonwealth government provides financial support for community and residential aged care services, and general practice which are predominantly privately owned (63). The Commonwealth government uses financial support to influence policy and practice in both aged care and primary health. During the COVID-19 pandemic for example, the Commonwealth government successfully used this power to leverage the Communicable Diseases Network Australia restrictions to protect the elderly in Residential Aged Care in the aged care industry (64). It has been supported by state governments in this. In other situations, however there have been disparities between Commonwealth recommendations and state restrictions based on assessment of the level of risk within a given state. For example, the Commonwealth has sought to use funding to encourage the earlier opening of private schools, which has been in contrast to public schools funded by State Governments who desired a longer shut-down period. This can result in public confusion and frustration if advice and actions are different. Further, local governments (councils) in two Australian states (Victoria and South Australia) have a legislative responsibility for public health creating a third level of governance (65). This can all impact on public trust because it brings complexity and uncertainty into play.

### Tailoring Public Health Messages

Brown notes that both health literacy and level of trust in public health officials vary according to social characteristics, leading to sub-groups who are more or less receptive to public health messages. He argues that it is “vital that we pay attention to different sub-groups in political systems, and acknowledge how varying experiences and perceptions of government and healthcare organizations, shaped at the intersections of class, gender, race and ethnicity, will shape very different relations and approaches to risk” ((14), p. 3). These groups may require different forms of communication to overcome negative perceptions of health organizations and their recommendations. Gray et al. found for example, that Pacific Islander peoples were more likely to trust information about H1N1 management received from community rather than other more official sources. They conclude that trust must be considered in tailoring health messages (22). There is also evidence from previous epidemics, that some population groups place greater trust in informal sources of information (such as social media) rather than formal sources such as public health organizations, resulting in greater levels of worry (16). Therefore, public health officials must consider whom they are communicating with and ensure that trust is formed with all members of the public.

### Personal Responsibility vs. Enforcement

A point of difference between pandemic management and food regulation is that failure to follow public health directives has implications beyond the individual and their immediate families. As such, there is greater legal and moral regulation of public health behaviors. Police in Australia have been given a range of powers to protect state boundaries; ensure restrictions on public gatherings; to enforce quarantine for people returning from overseas or who have been diagnosed with or in contact with someone diagnosed with COVID-19; to enforce closure of non-essential businesses and to limit access to residential aged care facilities (66). The extent to which legal enforcement is responsible for compliance with public health directives and how much is driven by trust in public health officials and information is unclear, although past epidemic literature suggests that the punitive measures are likely to play a role (50).

In contrast to these coercive approaches, behavior change may also be achieved through a form of public self-governance created through the moralization of public behavior. An example of this was described by researchers in Hong Kong, who found that appealing to nurses' moral responsibility to patients was effective in increasing uptake of influenza vaccinations (67). Conversely, moral discourses can be used as the basis for rejecting public health messages. Protesters against restrictions associated with COVID-19 in the United States and United Kingdom have drawn upon libertarian discourses of individual and religious freedoms from state interference to challenge ongoing restrictions (68). An alternative moral discourse, drawing on concerns for vulnerable members of our community, could counter this libertarian opposition to public health intervention.

## Conclusion

In this paper, we have highlighted the need to undertake research about global pandemics, specifically, the relationship between government action, public trust and public health behavior in the context of COVID-19. We applied our model, previously developed in the context of food safety incidents and scandals, to highlight some strategies that may apply to communicating effectively with the public around COVID-19 and public health behaviors that are required for the ongoing good health of the public. There are differences between food safety and pandemic management related to the ongoing role of food regulatory organizations in relationship development and establishment of communication strategies. Further, pandemic management requires widespread community rather than individual compliance for success. We emphasize that any research in risk mitigation and risk communication needs to be undertaken in different population groups because of the different needs different groups may have in terms of communication strategies, taking into account the actors involved, type of risks, and socio-political context of a pandemic response. Such research is important to ensure that public trust in the government and public health officials is maintained, government advice is followed, and the health of the public is maximized during current and future pandemics.

## Data Availability Statement

The original contributions presented in the study are included in the article/supplementary material, further inquiries can be directed to the corresponding author/s.

## Author Contributions

JH wrote the first draft of the paper and all authors provided a written contribution and approved the final version. AW, PW, JC, ET, DM, SM, and TW were involved in the development of the Model referred to in this paper. HP reviewed the literature used in this paper. AW provided leadership to the team. All authors contributed to the ideas explored in the paper.

## Conflict of Interest

The authors declare that the research was conducted in the absence of any commercial or financial relationships that could be construed as a potential conflict of interest.
